# Increased risk of ischemic stroke in patients with mild traumatic brain injury: a nationwide cohort study

**DOI:** 10.1186/s13049-014-0066-y

**Published:** 2014-11-19

**Authors:** Yi-Kung Lee, Chen-Wen Lee, Ming-Yuan Huang, Chen-Yang Hsu, Yung-Cheng Su

**Affiliations:** Emergency Department, Dalin Tzu Chi Hospital, Buddhist Tzu Chi Medical Foundation, No.2, Minsheng Rd, Dalin Township Chiayi County, 622 Taiwan; School of Medicine, Tzu Chi University, Hualien, Taiwan; Department of Emergency Medicine, Mackay Memorial Hospital, Taipei, Taiwan; Department of Public Heath, National Taiwan University, Taipei, Taiwan

**Keywords:** Stroke, Traumatic brain injury, National health insurance

## Abstract

**Background:**

It is known that the risk of stroke in patients with traumatic brain injury might be increased. However, the relationship between mild traumatic brain injury and ischemic stroke has never been established. We conducted a study of patients in Taiwan with mild traumatic brain injury to evaluate if they had a higher risk of stroke compared with the general population.

**Methods:**

We utilized a sampled National Health Insurance claims database containing one million beneficiaries. We followed all adult beneficiaries older than 18 years from January 1, 2007 to December 31, 2010 to determine if they were diagnosed with ischemic stroke. We further identified patients with mild traumatic brain injury and compared their risk of ischemic stroke with the general population.

**Results:**

We identified 24,905 patients with mild traumatic brain injury and 719,811 patients without mild traumatic brain injury. After controlling for age, gender, urbanization level, socioeconomic status, diabetes, hypertension, coronary artery disease, hyperlipidemia, history of alcohol intoxication, malignancies, heart failure, atrial fibrillation, smoking, obesity, epilepsy, peripheral artery disease and Charlson Comorbidity Index score, the adjusted hazard ratio for ischemic stroke was 1.46 (95% confidence interval, 1.33—1.62).

**Conclusion:**

Mild traumatic brain injury is an independent significant risk factor for ischemic stroke.

## Introduction

Each year, traumatic brain injury (TBI) accounts for 2.4 million emergency department visits, hospitalizations, or death in the United States, and the direct medical costs of TBI in the United States in 2010 were estimated to be $11.5 billion [1]. In many other countries, TBI also is one of the major causes of morbidity and mortality [2,3]. TBI can induce damage of varying extent because of brain swelling, axonal injury, hypoxia, inflammatory responses, oxidative stress and neurodegeneration [4,5]. Studies have also found that TBI may be associated with progression of some diseases, including epilepsy, Alzheimer’s disease, Parkinson’s disease, and psychiatric diseases [6-9].

Recently, in several studies TBI was found to be associated with increased risk of [10-12]. Because stroke results from disturbance of the blood supply to the brain, it has been hypothesized that vascular damage from TBI may also induce stroke in survivors [11]. It is well known that TBI can be classified into severe, moderate, and mild types based on the consciousness level, i.e., the Glasgow Coma Scale, and long-term sequelae are more common in patients with severe injuries [13,14]. In previous studies, we found that even in mild TBI, victims may still have higher risks of developing dementia and mortality [15,16]. However, the relationship between mild TBI and ischemic stroke has not yet been evaluated.

The aim of this study was to investigate the correlation between mild TBI and ischemic stroke by utilizing a large administration database. The results of this study might provide clinicians with further insights into this frequently encountered situation.

## Methods

### Ethics statement

This study was initiated after approval by the Institutional Review Board of Dalin Tzu Chi Hospital, Buddhist Tzu Chi Medical Foundation, Chiayi, Taiwan. Because all personal identification was stripped from the secondary files before analysis, the review board waived the requirement of obtaining written informed consent from the patients.

### Database

The National Health Insurance (NHI) program in Taiwan was implemented in 1995 and provides compulsory universal health insurance. It enrolls up to 99% of the Taiwanese population and has contracts with 97% of all medical providers [17]. The database contains comprehensive information on all insured individuals, including sex, date of birth, residential or work location, dates of clinical visits, the International Classification of Diseases (Ninth Revision) Clinical Modification (ICD-9-CM) diagnostic codes, details of prescribed medications, expenditure amounts and outcome at hospital discharge (i.e., recovered, died, or transferred out). A random sample consisting of one million people based on the 2005 reimbursement data was established for public access. This group did not significantly differ statistically from the larger cohort in age, gender, or health care costs according to the Taiwan National Health Research Institute. A sampled group was used as our study cohort.

### Study population

**Figure 1:**
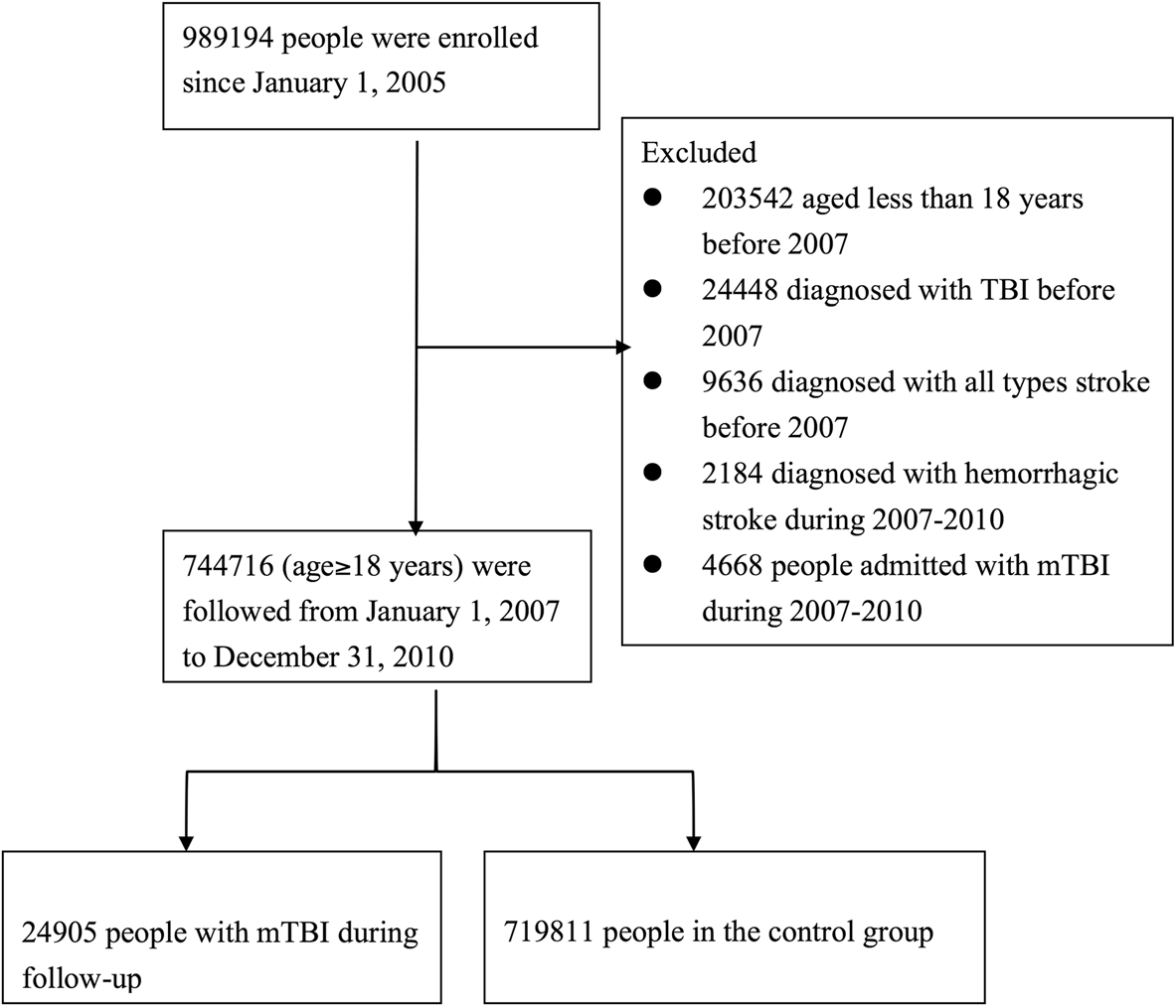
**Flow diagram of population-based study.**

The sampled population was followed for 6 years from January 1, 2005 to December 31, 2010. We identified individuals for our study cohort who were still alive in 2007 and were older than 18 years. Mild TBI was defined by the ICD-9-CM code for head concussion (850.0, 850.1, 850.5, or 850.9), intracranial injury of other and unspecified nature (854.0), or head injury, unspecified (959.01) [16,18]. Skull fracture (800.0, 800.5, 801.0, 801.5, 803.0, 803.5, 804.0, or 804.5) was not used as an alternative diagnosis because of the relative high impact of the injury [12]. Stroke was defined as either hemorrhagic (ICD-9-CM code 430–432) or ischemic (ICD-9-CM codes 433–437). To maximize case ascertainment, we only included patients hospitalized for stroke. We excluded patients with mild TBI and any type of stroke diagnosed before January 1, 2007. Since the ICD-9-CM codes for traumatic intracranial hemorrhage and hemorrhagic stroke may overlap, our study only focused on ischemic stroke. Patients who had been diagnosed with hemorrhagic stroke during the follow-up period were excluded. To avoid misclassification, we further excluded patients who had ever been hospitalized with TBI to ensure that enrolled patients with mild TBI were discharged directly after outpatient clinic or emergency departments visits. After exclusion of our cohort cases, we identified 24,905 patients with mild TBI and 719,811 without mild TBI. Each group was tracked from the date of mild TBI or January 1, 2007 (baseline) until December 31, 2010 (study end) to determine if ischemic stroke had been diagnosed during that period. Cases were censored for patients who were not beneficiaries anymore from the NHI Program (i.e., death or transfer out) or were still robust at the end of the follow-up period (Figure 1).

### Covariates

To better understand the effect of mild TBI on the risk of ischemic stroke, we used several covariates including patient demographics such as age, sex, urbanization level (i.e., urban, suburban, and rural areas) and socioeconomic status (SES). Income-related insurance payment amounts were used as a proxy measure of individual SES at follow-up. People were classified into three groups: (1) low SES: payment lower than US$571 per month (New Taiwan Dollars [NT$] 20,000); (2) moderate SES: payment between US$571–1141 per month (NT$ 20,000–40,000); and (3) high SES: US$1142 or more payment per month (NT$40,001) or more [15]. Also, the prevalence of selected comorbid conditions (i.e., diabetes, hypertension, coronary artery disease, hyperlipidemia, history of alcohol intoxication, malignancies, heart failure, atrial fibrillation, smoking, obesity, epilepsy, peripheral artery disease) and the Charlson Cormobidity Index (CCI) score were determined according to the discharge diagnosis either during outpatient clinic visits or hospitalizations before January 1, 2007. The CCI is a scoring system that includes weighing factors of important concomitant diseases; it has been validated for use with ICD-9-CM coded administrative databases [19,20].

### Statistical analysis

The SAS statistical package, version 9.2 (SAS Institute, Inc., Cary, NC, USA), and STATA version 11.2 (StataCorp, College Station, TX, USA) were used for data analysis. All covariates were taken as categorical variables except age, which was treated as a continuous variable. Categorical variables were compared with Pearson’s chi-square test and continuous variables with the t test to reveal the baseline heterogeneity in the two groups. The Nelson-Aalen cumulative hazard estimates were first plotted to show the trend of ischemic stroke. Cox proportional hazard regression models were then used to calculate the hazard ratios (HRs) of ischemic stroke for individuals with mild TBI after adjustments for age, gender, urbanization level, SES, diabetes, hypertension, coronary artery disease, hyperlipidemia, history of alcohol intoxication, malignancies, heart failure, atrial fibrillation, smoking, obesity, epilepsy, peripheral artery disease, and the CCI. The reason we chose Cox regression analyses is because it is more statistically powerful and efficient [21,22]. Adjusted HRs were analyzed both for 1) from mild TBI or baseline through study end, and 2) from 12 months after mild TBI or baseline through study end.

To further assess the robustness of our results, we performed a subgroup analysis to evaluate the risk of ischemic stroke in patients who had been hospitalized because of TBI to see if there is a ‘dose-response’ effect in the relationship between TBI and ischemic stroke. A two-tailed P value of <0.05 was considered significant.

## Results

**Figure 2:**
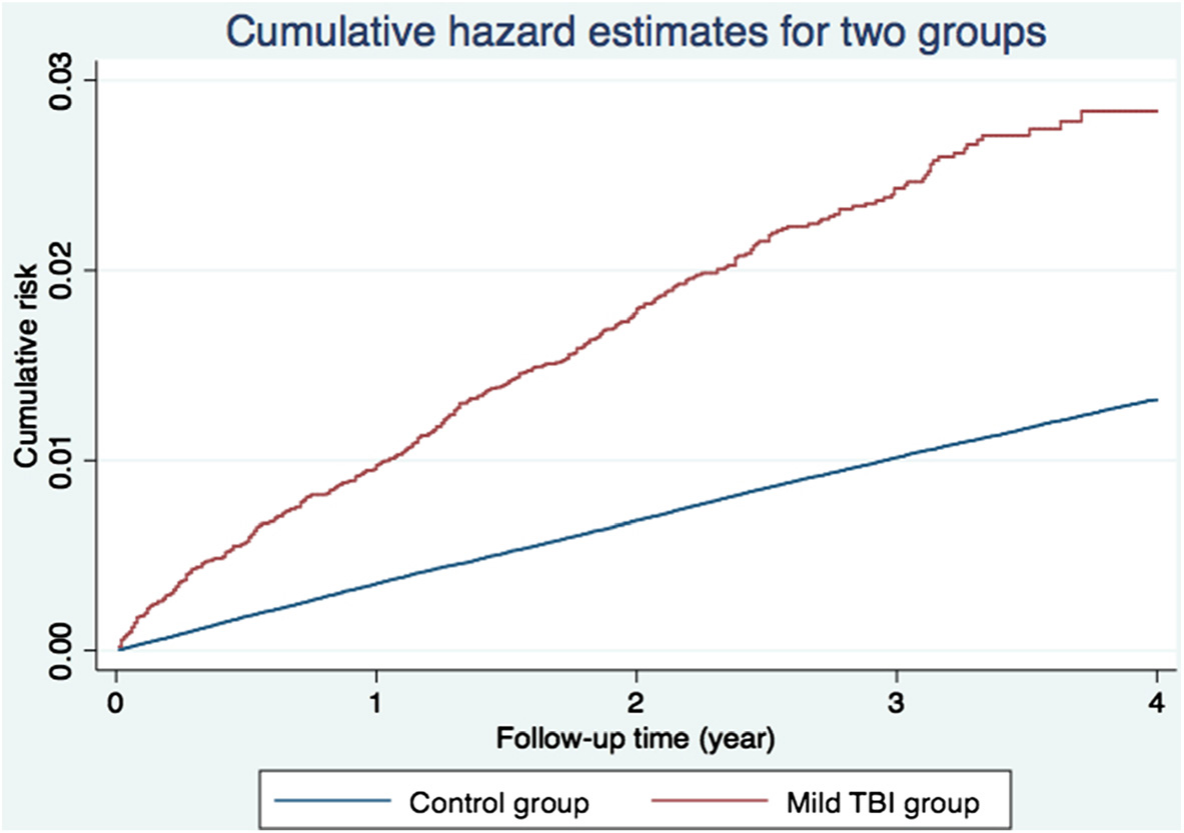
**Nelson-Aalen plot shows higher cumulative risk of ischemic stroke in the mild TBI group.**

The distribution of both demographic characteristics and selected clinical characteristics is shown in Table 1. There were 24,905 patients in the mild TBI group and 719,811 in the control group. Total follow-up periods in the two groups were 48,371 and 2,793,892 person-years, respectively. In the mild TBI group 13.7% of patients underwent computed tomography (CT). Patients with mild TBI were significantly older and had significantly more comorbidities than the controls. By the end of follow-up, 9,654 patients had been diagnosed with ischemic stroke, including 412 in the mild TBI group and 9,242 in the control group. The average duration from mild TBI to ischemic stroke was 1.12 years (95% confidence interval [CI], 1.03—1.20). The crude HR for ischemic stroke between the two groups was 2.49 (95% CI, 2.25—2.74). Nelson-Aalen plot curves showed a higher trend for ischemic stroke in the mild TBI group (Figure 2).

**Table 1:** **Baseline demographic and clinical of the mild TBI group and control group**

**Variables**	**Mild TBI group**		**Control group**		***P*** **-value**
	**(n = 24,905)**		**(n = 719,811)**		
	**No.**	**%**	**No.**	**%**	
Male	11814	47.4	348981	48.5	**0.001**
Mean age (SD)	46.1	20.1	43.5	16.3	**<0.001**
Mean follow-up year (SD)	1.94	1.18	3.88	0.55	
Socioeconomic status					**<0.001**
Low	12765	51.3	306667	42.6	
Moderate	9802	39.4	290800	40.4	
High	2338	9.4	122344	17.0	
Urbanization level					**<0.001**
Urban	6867	27.6	220594	30.7	
Suburban	11445	46.0	329139	45.7	
Rural	6593	26.5	170078	23.6	
Diabetes	2466	9.9	51555	7.2	**<0.001**
Hypertension	4936	19.8	111872	15.5	**<0.001**
Coronary artery disease	3878	15.6	78417	10.9	**<0.001**
Hyperlipidemia	2849	11.4	71010	9.9	**<0.001**
History of alcohol intoxication	479	1.9	5133	0.7	**<0.001**
Malignancies	847	3.4	21913	3.0	**0.001**
Charlson Comorbidity Index score					**<0.001**
0	14794	59.4	490058	68.1	
1	5791	23.3	140609	19.5	
≥ 2	4320	17.4	89144	12.4	
Heart failure	508	2.0	8294	1.2	**<0.001**
Atrial fibrillation	133	0.5	2654	0.4	**<0.001**
Smoking	252	1.0	5412	0.8	**<0.001**
Obesity	106	0.4	2359	0.3	**0.008**
Epilepsy	407	1.6	3919	0.5	**<0.001**
Peripheral artery disease	244	1.0	4008	0.6	**<0.001**

Statistical results with p<0.05 were presented in boldface.

Next, we performed the multivariate Cox regression models to evaluate the adjusted HRs of ischemic stroke. Patients with mild TBI still had higher HRs after controlling for age, gender, urbanization level, SES, diabetes, hypertension, coronary artery disease, hyperlipidemia, history of alcohol intoxication, malignancies, heart failure, atrial fibrillation, smoking, obesity, epilepsy, peripheral artery disease and CCI score (1.46; 95% CI, 1.33—1.62). Other independent risk factors of ischemic stroke included older age, male gender, living outside of an urban area, lower SES, diabetes, hypertension, coronary artery disease, history of alcohol intoxication, heart failure, atrial fibrillation, epilepsy and higher CCI. The statistical results are summarized in Table 2.

**Table 2:** **Adjusted HRs of ischemic stroke for patients followed from baseline to study end**

**Mild TBI group (n = 24,905)**	**Control group (n = 719,811)**		
**Variables**	**Hazard ratio**	**95% confidence interval**	**P-value**
Mild TBI	1.46	1.33—1.62	**<0.001**
Male	1.47	1.41—1.53	**<0.001**
Patient age	1.07	1.06—1.07	**<0.001**
Socioeconomic status			
Low	1	--	--
Moderate	0.80	0.76—0.84	**<0.001**
High	0.49	0.44—0.55	**<0.001**
Urbanization level			
Urban	1	--	--
Suburban	1.17	1.11—1.23	**<0.001**
Rural	1.39	1.32—1.48	**<0.001**
Diabetes	1.57	1.49—1.65	**<0.001**
Hypertension	1.77	1.68—1.85	**<0.001**
Coronary artery disease	1.43	1.37—1.50	**<0.001**
Hyperlipidemia	0.89	0.84—0.93	**<0.001**
History of alcohol intoxication	1.46	1.20—1.76	**0.001**
Malignancies	1.06	0.99—1.14	0.122
Charlson Comorbidity Index score			
0	1	--	--
1	1.37	1.29—1.45	**<0.001**
≥ 2	1.57	1.47—1.67	**<0.001**
Heart failure	1.11	1.03—1.21	**0.008**
Atrial fibrillation	1.42	1.26—1.60	**<0.001**
Smoking	0.99	0.79—1.24	0.937
Obesity	1.13	0.83—1.56	0.433
Epilepsy	2.13	1.85—2.45	**<0.001**
Peripheral artery disease	1.06	0.93—1.21	0.384

Statistical results with p<0.05 were presented in boldface.

An analysis based on individuals who survived longer than 12 months was performed. There were 18,092 patients in the mild TBI group and 708,879 controls. The HR for mild TBI was slightly decreased but still statistically significant (1.38; 95% CI, 1.20—1.59) (Table 3).

**Table 3:** **Adjusted HRs of ischemic stroke for patients who survived longer than 12 months**

**Mild TBI group (n = 24,905)**	**Control group (n = 719,811)**		
**Variables**	**Hazard ratio**	**95% confidence interval**	**P-value**
MildTBI	1.38	1.20—1.59	**<0.001**
Male	1.49	1.42—1.56	**<0.001**
Patient age	1.07	1.06—1.07	**<0.001**
Socioeconomic status			
Low	1	--	--
Moderate	0.84	0.79—0.89	**<0.001**
High	0.53	0.47—0.61	**<0.001**
Urbanization level			
Urban	1	--	--
Suburban	1.15	1.08—1.22	**<0.001**
Rural	1.36	1.27—1.46	**<0.001**
Diabetes	1.57	1.48—1.67	**<0.001**
Hypertension	1.74	1.64—1.84	**<0.001**
Coronary artery disease	1.46	1.39—1.54	**<0.001**
Hyperlipidemia	0.90	0.85—0.96	**0.001**
History of alcohol intoxication	1.27	1.00—1.62	**0.050**
Malignancies	1.06	0.97—1.16	0.191
Charlson Comorbidity Index score			
0	1	--	--
1	1.32	1.23—1.41	**<0.001**
≥ 2	1.46	1.36—1.57	**<0.001**
Heart failure	1.11	1.00—1.22	**0.041**
Atrial fibrillation	1.47	1.27—1.69	**<0.001**
Smoking	1.14	0.89—1.46	0.314
Obesity	1.28	0.91—1.82	0.161
Epilepsy	2.04	1.71—2.42	**<0.001**
Peripheral artery disease	1.09	0.93—1.27	0.305

Statistical results with p<0.05 were presented in boldface.

A subgroup analysis based on patients admitted with TBI was conducted. There were 4,668 patients in the TBI group and 719,811 in the control group. After controlling for the same covariates, the HR for ischemic stroke in the hospitalized group was higher than for those discharged directly after visits (HR 3.43; 95% CI, 2.97—3.95). The statistical results of other covariates were similar to those of the primary study cohort and are summarized in Table 4.

**Table 4:** **Adjusted HRs of ischemic stroke for patients admitted with TBI**

**Variables**	**Hazard ratio**	**95% confidence interval**	**P-value**
Mild TBI	3.43	2.97—3.95	**<0.001**
Male	1.46	1.40—1.52	**<0.001**
Patient age	1.07	1.06—1.07	**<0.001**
Socioeconomic status			
Low	1	--	--
Moderate	0.81	0.77—0.85	**<0.001**
High	0.50	0.45—0.56	**<0.001**
Urbanization level			
Urban	1	--	--
Suburban	1.17	1.11—1.23	**<0.001**
Rural	1.38	1.30—1.46	**<0.001**
Diabetes	1.58	1.50—1.66	**<0.001**
Hypertension	1.77	1.69—1.86	**<0.001**
Coronary artery disease	1.43	1.37—1.49	**<0.001**
Hyperlipidemia	0.88	0.84—0.93	**<0.001**
History of alcohol intoxication	1.46	1.20—1.77	**<0.001**
Malignancies	1.07	0.99—1.15	0.092
Charlson Comorbidity Index score			
0	1	--	--
1	1.37	1.29—1.45	**<0.001**
≥ 2	1.55	1.46—1.65	**<0.001**
Heart failure	1.12	1.04—1.22	**0.004**
Atrial fibrillation	1.42	1.26—1.61	**<0.001**
Smoking	1.00	0.80—1.25	0.984
Obesity	1.00	0.71—1.40	0.995
Epilepsy	2.06	1.78—2.38	**<0.001**
Peripheral artery disease	1.10	0.96—1.26	0.170

Statistical results with p<0.05 were presented in boldface.

## Discussion

Studies have found that TBI is associated with increased risk of stroke [10-12]. Although the actual mechanism is still not completely understood, several hypotheses have been proposed. Impaired blood supply after damage to the cerebrovascular system caused by TBI might induce a stroke. Alterations in the coagulation cascade, clot formation and free radical generation after TBI may also be explanations [11,23,24]. Moreover, elevated intracranial pressure and blood pressure commonly noted among TBI patients may lead to stroke [25]. In our study, we further extended investigation of the impact of TBI to the mild type, utilizing a large administrative cohort and found that patients with a single mild TBI have a higher risk of ischemic stroke later in their lives compared to the general population. Our study had enough statistical power to provide a precise estimate of the HR (1.46; 95% CI, 1.33—1.62), which was statistically and clinically significant. The database corresponds well to the whole population; therefore, loss of follow-up or selection bias were not concerns. In Chen *et al*., the adjusted HR for stroke in all TBI patients who survived for more than one year was 4.61 (95% CI, 4.16 –5.11) [11]. Compared to their results, HRs in our two mild TBI groups were lower, indicating that the degree of impact may be a factor in the occurrence of different sequelae.

Another advantage of our study is that we directly compared mild TBI patients with the general population simultaneously by survival analysis. Using this design, we were able to adjust extensively for possible confounding factors. Among the covariates, older age, male gender, living outside of an urban area, lower SES, diabetes, hypertension, coronary artery disease, history of alcohol intoxication, heart failure, atrial fibrillation, epilepsy and higher CCI scores were found to be associated with higher risks of ischemic stroke in patients with TBI, which is consistent with previous publications [12,26-28].

This study had several limitations. First, our findings were derived from administrative data. Cases were collected using ICD-9-CM diagnosis codes, a relatively outdated system which is good for insurance reimbursement but is not a substitute for precise operative definition. Therefore, the validity of the diagnosis (i.e., sensitivity, specificity and accuracy) was not fully assessed. In Bazarian et al. [18], the sensitivity of ICD-9-CM codes for mild TBI was 45.9% with a specificity of 97.8%. In other words, patients in the mild TBI group were highly likely to have mild TBI, while some individuals in the control group may had mild TBI during the study period but the inclusion strategy failed to identify them. In this situation, the predicted effect of mild TBI on ischemic stroke should be toward the null, but we still found a significant risk of ischemic stroke in patients with mild TBI.

Second, we were unable to obtain the clinical information for patients with mild TBI, such as the Glasgow Coma Scale score, findings on cranial CT, the injury mechanism and the initial presentations. By definition, labeling our cases as ‘mild TBI’ may have been inappropriate. However, it has been validated that the ICD9-CM codes have high specificity regarding diagnosis of mild TBI [18]. Furthermore, we excluded those patients who were admitted to the hospitals to make sure that the patients enrolled were really in the “mild” category. Based on our inclusion criteria, although not totally precise, we think the cases in our study group were highly correlated with the definition of mild TBI [16].

Third, although we extensively adjusted for possible comorbidities, unmeasured cofounding is still an issue. However, we did a subgroup analysis and found a higher HR of ischemic stroke (3.43) in patients admitted with TBI and the unmeasured confounding could not fully explain the ‘dose-response’ of injury severity.

## Conclusions

Mild TBI is an independent significant risk factor for ischemic stroke. The results indicate that more emphasis on head injury prevention and public awareness about long term sequelae of TBI would be worthwhile.
